# Greater Reduction in Abdominal Than in Upper Arms Subcutaneous Fat in 10- to 12-Year-Old Tennis Players: A Volumetric MRI Study

**DOI:** 10.3389/fped.2019.00345

**Published:** 2019-08-20

**Authors:** Joaquín Sanchis-Moysi, José Antonio Serrano-Sánchez, Juan José González-Henríquez, José A. L. Calbet, Cecilia Dorado

**Affiliations:** ^1^Research Institute of Biomedical and Health Sciences (IUIBS), Las Palmas de Gran Canaria, Spain; ^2^Department of Physical Education, University of Las Palmas de Gran Canaria, Las Palmas de Gran Canaria, Spain; ^3^Department of Mathematics, University of Las Palmas de Gran Canaria, Las Palmas de Gran Canaria, Spain; ^4^School of Kinesiology, University of British Columbia, Vancouver, BC, Canada; ^5^Department of Physical Performance, Norwegian School of Sport Sciences, Oslo, Norway

**Keywords:** adipose tissue, children, MRI, tennis, subcutaneous fat, abdominal fat

## Abstract

**Background:** Little is known about the impact of long term participation in sports and subcutaneous fat volume in children. This study aimed at determining whether tennis participation is associated with lower subcutaneous adipose tissue volume (SATv) in the abdominal and upper extremities in children.

**Methods:** Magnetic resonance imaging (MRI) was used to determine the SATv stored in the abdominal region and upper arms in seven tennis players and seven inactive children matched by height and age (147 cm and 10.9 years). All participants were in Tanner stage 1 or 2.

**Results:** Playing tennis was associated with 48% (*P* = 0.001) lower abdominal SATv and 17–18% (*P* > 0.05) lower upper arms SATv compared to controls. The ratio between abdominal/upper arms SATv was larger in the controls vs. tennis players (69% *P* = 0.001). The SATv was similar in the dominant and non-dominant arm within each group.

**Conclusion:** Playing tennis during childhood is associated with reduced SATv in the abdominal region and a more favorable regional distribution of fat. Despite the large amount of contractile activity of the playing (dominant) arm, there was no indication of between-arms differences in SATv.

## Introduction

Accumulation of subcutaneous adipose tissue (SAT) has important implications for health (1) and physical fitness (2, 3). Cardiometabolic risk and metabolic syndrome have been associated with increased accumulation of SAT of the upper body in humans (4). It is well-documented that exercise training may help reducing total adipose tissue stores (5). However, little is known about the effects of exercise training on the regional distribution of SAT in healthy adults (6), while children have not been studied.

Tennis is one of the most popular sports. During tennis practice, the lipolysis is strongly stimulated (7) what may contribute to explain the lower fat mass of tennis players (TPs) (8). Studies using dual-energy X-ray absorptiometry (DXA) showed that TPs of different ages and gender have a lower percentage of total body fat than healthy sedentary controls, mostly due to lower accumulation of fat mass in the trunk (8–10). In children, an inverse association between the number of years playing tennis and the percentage of total body fat has been reported, suggesting that long-term participation in tennis may further reduce fat mass (9). What remains to be determined is whether long-term tennis playing is associated with a specific regional reduction of fat mass.

The literature about the loss of SAT in a particular region of the body as a consequence of exercising that specific site (spot reduction) is scarce (11–16). Tennis offers an interesting experimental model to investigate the local effects of prolonged participation in sports on the subcutaneous mass, by comparing the dominant to the non-dominant arm. Tennis players submit the dominant arm, i.e., the arm holding the racket during the tennis serve, to an enormous amount of exercise compared to the contralateral. This asymmetric loading cause between-arm differences in muscle and bone morphology (17, 18) and maybe in the regional deposition of SAT in the upper extremity (19, 20). In previous studies using DXA, we observed a lower percentage of fat in the dominant compared to the non-dominant arm in professional TPs (8), but no such asymmetry was observed in children TPs (9). The use of magnetic resonance imaging (MRI) for the quantification of adipose tissue may help to ascertain whether tennis-playing induces spot reductions in adipose mass (21).

Magnetic resonance imaging and computed tomography (CT) are considered the reference standards for measuring adipose tissue (21). Both methods allow the volumetric quantification of SAT in the extremities and to distinguish SAT from visceral adipose tissue in the trunk (21, 22). DXA may also differentiate visceral from subcutaneous fat in the abdominal region, although with lower precision than MRI and CT (23). Ultrasonography allows measuring the thickness of the SAT but is less reliable and precise than MRI and CT (24, 25). Other techniques such as bioelectrical impedance analysis, anthropometric measurements (body mass index, waist-to-hip-ratio, waist circumference, or skinfold thickness) provide indirect estimations of regional fat distribution (24).

Few studies have analyzed the subcutaneous adipose tissue volume (SATv) in children using MRI (26, 27). Previous reports using MRI have based their conclusions on a single axial anatomical cross-sectional image (SAT area) to estimate the whole SAT in an entire anatomical region (28). However, a recent study in children has shown that an accurate assessment of total abdominal SATv requires multiple cross-sectional images (4).

The main purpose of the present study was to describe the distribution of SAT in the upper body (upper arms and abdominal region) in young TPs submitted to chronic tennis training. A secondary aim was to determine the differences in the regional distribution of SAT between the TPs and inactive healthy control children. The main hypothesis to be tested is that the TPs accumulate less SAT in the abdominal and upper arms regions compared to controls. We also hypothesized that the amount of SAT is lower in the dominant than the contralateral arm of TPs.

## Materials and Methods

### Design

A matched retrospective cohort design (tennis players vs. inactive) was followed. The sample size was determined to capture SATv reductions in tennis players vs. inactive of at least 40%, with a statistical power of 80% and an alpha error (one-sided) of 0.05. Previous studies had shown that the coefficient of variation (standard deviation/mean) of the SATv in children and adolescents fluctuated between 22 and 26% (26, 27). We adopted a more conservative position assuming a coefficient of variation of 30% (29). Also, given the matched-pairs of the participants, we assumed a zero correlation in the SATv between tennis players and controls. Thus, the appropriate sample size to capture a reduction of at least 40% of SATv in tennis players with at least 2 years of training and competition compared to inactive matched-pairs was of 14 participants (seven tennis players and seven controls). We assumed a coefficient of variation of SATv in children close to 30% and zero correlation between both groups. The statistical power was set at 80% and the one-sided alpha error at 0.05.

**Figure 1:**
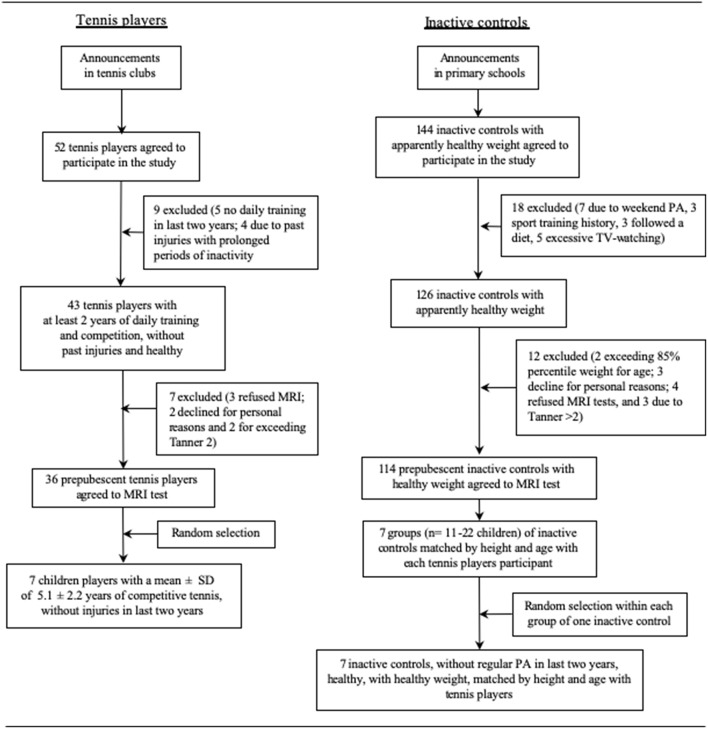
Flow chart for the selection of children tennis players and inactive controls.

The tennis players and controls were matched for height and age. For each tennis player selected, an inactive control child was chosen randomly from among those controls with <1 cm difference in height and <1 year of difference in age. Figure 1 describes the process.

### Participants

Fourteen children, seven TPs and seven controls, all males, in Tanner stage 1–2 participated in the study. To be included, all participants had to be healthy, without any chronic diseases and free of musculoskeletal conditions. The TPs were consecutively recruited from tennis clubs of Gran Canaria. Tennis players were selected randomly according to the following inclusion criteria: (1) they had started playing tennis before the age of nine (mean starting age, 6.3 ± 2.6 years); (2) had been participating in competitive tennis for a minimum of 2 years (mean 5.1 ± 2.2); and (3) trained at least 5 times per week (12.0 ± 2.1 h per week) excluding competitions. The TPs were not involved in any other regular form of exercise apart from the physical education compulsory sessions included in the Spanish academic curriculum (2 weekly sessions of 45 min each). The inactive children (control group) were recruited through local announcements in primary schools, among children who did not participate in any regular form of exercise, apart from the compulsory physical education curriculum, without sport training antecedents or excessive TV watching (>4 h/day). Of the 144 inactive children agreeing to participate, 18 were excluded due to physical activities during the weekends, excessive TV-watching, or dieting to lose weight. Another 12 children were excluded due to personal reasons, excess of weight according to CDC tables (30), or Tanner stage >2 (Figure 1). From the remaining 126 children, seven were selected randomly for MRI examination. Height and body mass were measured before MRI scanning, as described elsewhere (31). As expected, this resulted in the inactive children having a slightly higher BMI than the TPs (*P* = 0.06).

In the TPs, the arm holding the racket during the service stroke was considered as the dominant arm, whereas in the controls, it was the preferred arm used to perform an overhead throw. Two children played a two-handed backhand. Each participant and parents of children were informed about the aims and procedures of the study and gave their informed written consent to participate. The study was approved by the ethical committee of the University of Las Palmas de Gran Canaria.

### Pubertal Status Assessment

Tanner pubertal status was self-assessed with parental guidance using the standard five scale Tanner stages (32).

### Magnetic Resonance Imaging

**Figure 2:**
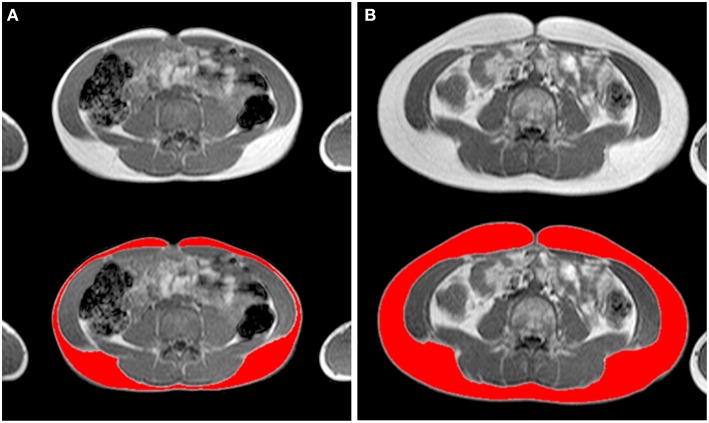
Axial T1-weighted in-phase non-fat saturated magnetic resonance images at L4 level illustrating the subcutaneous adipose tissue of **(A)** a child tennis player and **(B)** an inactive child (weight 35.6 vs. 41.7 kg, height 148.1 vs. 147.5 cm, age 11.0 vs. 10.2 years, respectively, both Tanner stage 2), matched for height and age. Top, gray-scale images; bottom, corresponding analyzed images (in red, the subcutaneous adipose tissue) (Supplementary Material).

**Figure 3:**
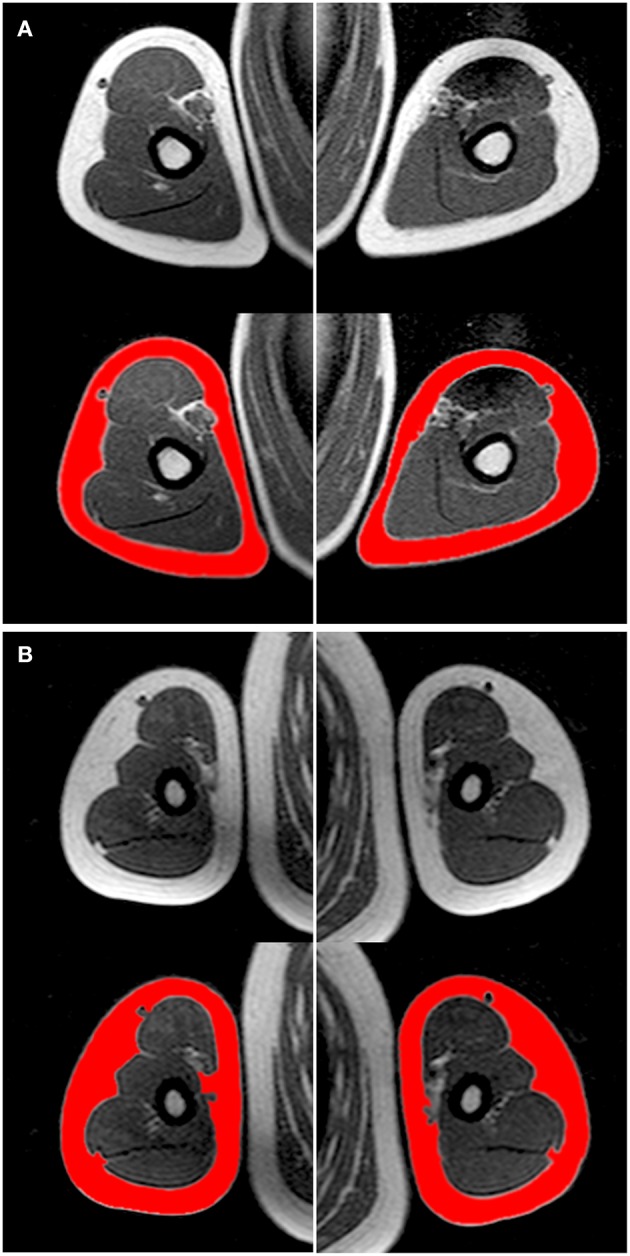
Axial T1-weighted in-phase non-fat saturated magnetic resonance images at the level of the mid-arm, illustrating the subcutaneous adipose tissue of **(A)** a child tennis player and **(B)** an inactive child (weight 35.6 vs. 41.7 kg, height 148.1 vs. 147.5 cm, age 11.0 vs. 10.2 years, respectively, both Tanner stage 2), matched for height and age. Top, gray-scale images; bottom, corresponding analyzed images (in red, the subcutaneous adipose tissue) (Supplementary Material).

A 1.5 T MRI scanner (Philips Achieva 1.5 Tesla system, Philips Healthcare, Best, The Netherlands) was used to acquire 8-mm contiguous slices (without interslice separation) from the trunk and 10-mm axial contiguous slices from each upper extremity independently. The subjects were examined in the supine position with arms lying parallel along the lateral sides of the body. Sagittal, coronal, and transverse localizers of the regions of interest were used to determine the anatomic sites for image acquisition precisely. A body coil was used to obtain transverse MR images perpendicular to the anterior abdominal wall at rest, while the subjects hold their breath at mid expiration. The total exploration time for the trunk was about 20 s, which was within the breath-hold tolerance of all subjects. Axial gradient-echo T1-weighted in-phase and out-of-phase MR images were acquired for the trunk (repetition time: 112 ms; echo time: 4.2 ms; flip-angle: 80°; field of view: 42-cm^2^; matrix: 256 × 256 pixels, in-plane spatial resolution 1.64 × 1.64 mm) (33) and the upper extremity (repetition time: 820 ms; echo time: 20 ms; field of view: 35-cm^2^; matrix: 512 × 512 pixels, in-plane spatial resolution 0.68 × 0.68 mm) (34). All sequences were obtained with no fat saturation. The MRI images were transferred to a computer for digital reconstruction to determine the subcutaneous fat cross-sectional areas (Figures 2, 3).

The total volume of SAT in the abdominal region was calculated from T12-L1 intervertebral disc to pubic symphysis. The SATv of each upper extremity was measured from the slice that immediately followed the axilla (the first image showing a gap between the arm and trunk) (16) to the more proximal image of the medial epicondyle.

All calculations were carried out by the same investigator using a specially designed image analysis software (SliceOmatic 4.3, Tomovision Inc., Montreal, Canada), as described elsewhere (35, 36). The investigator manually traced all images. A total of 1,150 slices (between 20 and 30 per body region and participant) were processed to determine the volume of SAT in the abdominal region and both arms. A threshold was selected for adipose and lean tissues using gray-level image pixel histograms to identify the tissue boundaries (35, 36).

The reliability of the assessment of SATv was determined by analyzing 10–20 slices from the upper arms and 20–30 slices from the abdominal region twice in all subjects, at least 2 weeks apart. The examiner was blinded regarding the previous results. The percentual variation of the assessments of the trunk and upper arms subcutaneous fat volumes were all below 1.5%.

### Statistical Analysis

The SATv variables were checked for normality and homoscedasticity by the Shapiro-Wilk and Kolmogorov-Smirnov tests with the Lilliefors correction. All the SATv variables adjusted well to the normality tests except the ratio of SAT abdomen/arms in the inactive group, which was logarithmically transformed. Both groups also presented homogeneous variances in all SAT variables (volumes, abdomen/arms ratio and asymmetry between arms, P Levene test between 0.44 and 0.89).

Mean, and the standard error of the mean (SE) are given as descriptive statistics. Differences between TPs and controls were tested using a mixed linear model (37). The comparisons were adjusted for BMI to compensate for the difference of 2 kg/m^2^ between both groups. Significant differences for SATv were assumed at *P* < 0.05 (one-sided). Lme4 and Lsmeans modules of R statistical package software were used for the analysis (37, 38). A priori sample size and *post-hoc* power were determined using G^*^Power software (39).

## Results

Table 1 summarizes the main characteristics of each group. The TPs had a slightly lower body mass index (BMI) compared to controls, but the difference was not statistically significant (*P* = 0.06).

**Table 1:** Physical characteristics of the tennis players and inactive controls.

	**Children**	
	**Tennis (*n* = 7)** **Mean ± SD**	**Controls (*n* = 7)** **Mean ± SD**
Age (years)	10.9 ± 0.7	10.9 ± 0.6
Height (cm)	147.3 ± 5.5	147.3 ± 5.1
Weight (Kg)	37.4 ± 6.2	42.1 ± 4.5
BMI (Kg/m^2^)	17.1 ± 1.9	19.4 ± 2.2

**Figure 4:**
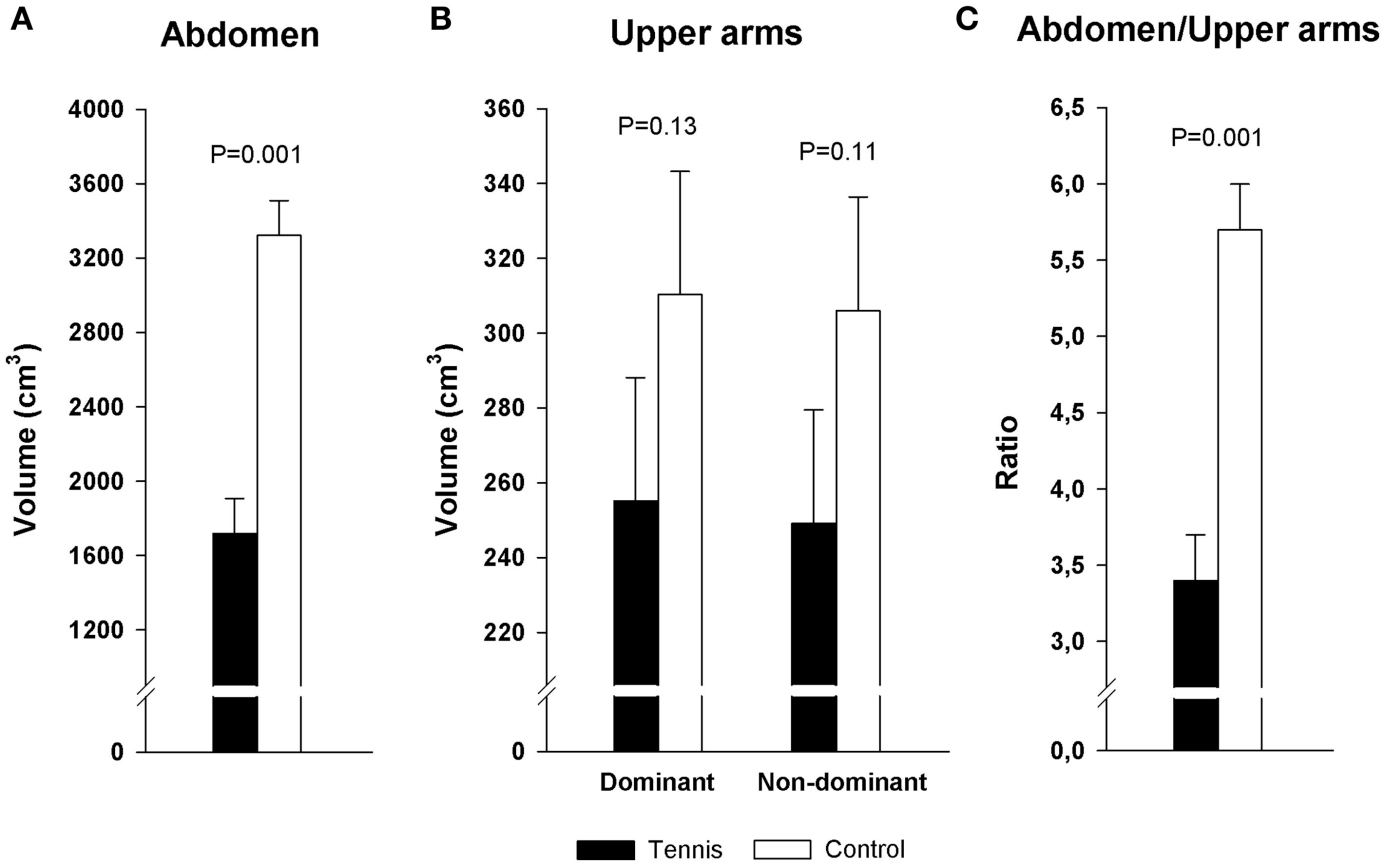
Volume of subcutaneous adipose tissue in tennis players and inactive controls **(A)** in the abdominal region, **(B)** in the upper arms, **(C)** in the ratio abdomen/arms. Error bars represent the standard error of mean.

The TPs had 48% lower volume of SAT (mean ± SE) in the abdominal region compared to their inactive counterparts (1,718 ± 187 vs. 3,322 ± 187 cm^3^, *P* = 0.001; Power = 1.00; Figure 4A).

The TPs had 17 and 18% lower SAT than the controls in the dominant (255 ± 33 vs. 310 ± 33 cm^3^, *P* = 0.134) and non-dominant arms (249 ± 30 vs. 306 ± 30 cm^3^, *P* = 0.106), respectively (Figure 4B). The *post-hoc* statistical power for these differences was 0.24–0.28.

The TPs presented a different distribution of SAT compared to controls. The abdomen/arms ratio was 40.8% lower than controls (3.4 ± 0.3 vs. 5.7 ± 0.3 cm^3^, in the TPs and controls, respectively, *P* = 0.001, power = 1.00; Figure 4C).

When the volume of SAT of the dominant arm was compared with the contralateral arm into each group, no significant differences were observed in either group (2.0 ± 2.5 and 0.39 ± 2.5 in TP and controls, respectively, *P* = 0.212, power = 0.10).

## Discussion

In the present study, the TPs had 48% lower SATv in the abdominal region and 17–18% lower SATv in upper arms compared to height-matched inactive healthy children. The abdomen/arms ratio was substantially lower in TPs, supporting regional differences in fat storage associated with the practice of tennis in prepubertal children. The fact that no SAT differences were observed between dominant and non-dominant arms in either group (SAT asymmetry) is compatible with preferential utilization of SAT from the abdominal region than from the upper arms in TPs.

Excessive accumulation of SAT in the abdominal region has been associated with a higher risk of metabolic and coronary diseases in pubertal age (4, 40, 41). It has been proposed that targeting the abdominal SAT with exercise training might help reducing abdominal SAT, and therefore, this could be an interesting clinical goal in children and adolescents (42). To the extent of our knowledge, few studies had investigated the effects of exercise training on abdominal SAT depots in children using state of the art technology, all them in obese (27, 43, 44). In a pioneer study using MRI, Owens et al. (27) observed that 16 weeks of playing games combined with exercises on machines (40 min each training session, at 70–75% of maximal heart rate, 5 sessions per week) did not decrease significantly (1%) the abdominal SAT in children 7–10 years old. In children of similar age studied by Davis et al. (43) with MRI, abdominal SAT decreased 6–9% after 13 weeks of aerobic training (20–40 min per training session, 5 sessions per week). Also using MRI, similar improvements in abdominal SAT (3–10%) have been reported in obese adolescents after varied exercise training programs of different durations (26, 45, 46). The large difference in abdominal SAT between our TPs and their control counterparts observed here suggests that long-term tennis playing may be an excellent stimulus to prevent or reduce abdominal SAT accumulation. This concurs with a recent study indicating that active lifestyles but not a short-term training intervention elevate mitochondrial content and oxidative markers in abdominal SAT (47).

A previous study using DXA in children TPs and controls, who had anthropometric characteristics and age similar to those participating in the present study, showed that the TPs that started in tennis before puberty had lower total body fat compared to age-matched inactive counterparts with normal weight (8, 9). Interestingly, the difference in total body fat observed in the cited study was much lower than the difference in abdominal SAT reported here (23 vs. 48%, respectively) (9). This highlights the plasticity that abdominal SAT has compared to other fat depots in response to regular exercise.

It is well-established that in the general population total body SAT increases from prepuberty to adolescence; however, abdominal SAT remains stable in the transition from pre-to-puberty (28). It has been suggested that during this transition period SAT is growing in other areas different than the abdominal region, i.e., the extremities (48). Our results support this hypothesis and suggest a preferential mobilization of SAT from the abdominal region than from the upper arms in young TPs who are starting sexual maturation (10–12 years old, Tanner stage 2).

The literature about whether exercising a specific part of the body may elicit a local loss of SAT (spot reduction) is scarce and conflicting (11–16, 21). Regional specificity in the lipolytic response to exercise may be due to differences in the distribution of adrenergic receptors between adipose areas (49). Our study shows for the first time using MRI that despite the high amount of exercise performed by the dominant arm, playing tennis does not elicit a differential adaptation in the volume of SAT compared to the contralateral arm, in young TPs. This concurs with a pioneer study using MRI showing spot reductions in the upper extremity did not occur as a result of unilateral resistance training in adults (16). On the other hand, our findings are also in agreement with physiological studies analyzing the adaptations in the SAT close to the working muscles. These studies indicate that the SAT does not appear to be used to supply energy substrates to the neighboring muscles (50, 51).

The present study should be interpreted with caution since it was conducted in a small sample of TPs and controls. The absence of significant differences in the upper arms was probably due to the small sample size. However, the differences found in the abdominal region and the ratio abdomen/arms were consistent and observed in children that have been practicing competitive tennis for an average of 5 years. The participants in our study had similar anthropometric characteristics compared to the general population of Gran Canaria matched for age and sex (31). Nevertheless, we cannot rule out differences due to the potential influence of genetic, nutritional, or environmental factors on SATv stores (28). Another limitation is that pubertal staging may have been biased by parents assessment (52). Due to the small amount of SATv in the TPs, it was not possible to distinguish deep from superficial SATv. Deep SAT has been associated with metabolic abnormalities (53) and should be examined in future studies.

In conclusion, the present study shows that long-term tennis playing during childhood is associated with a lower accumulation of SATv in the abdominal region compared to age- and height-matched healthy inactive children. These differences were higher in the abdomen than in upper arms suggesting a preferential utilization of SAT from the abdominal region in the TPs. Finally, this study shows that no spot-reductions in SATv occur in the dominant compared to the non-dominant arm of TPs despite vast differences in loading.

## Data Availability

The dataset generated for this study is provided as Supplementary Material.

## Ethics Statement

This study was carried out in accordance with the recommendations of the ethic committee of the University of Las Palmas de Gran Canaria. Each participant and parents of children were informed about the aims and procedures of the study and gave their written informed consent to participate in accordance with the Declaration of Helsinki. The protocol was approved by the ethic committee of the University of Las Palmas de Gran Canaria.

## Author Contributions

JS-M, JS-S, and CD contributed to the conception and design of the study. JC and CD prepared and selected the sample. JS-M delineated the MRI images and wrote the first draft of the manuscript. JS-S and CD reviewed the traced images. JS-M and JS-S calculated the SATv and designed the database. JS-M and JG-H performed the statistical analysis. JS-S, JC, and CD wrote sections of the manuscript. All authors contributed to manuscript revision, read and approved the submitted version.

### Conflict of Interest Statement

The authors declare that the research was conducted in the absence of any commercial or financial relationships that could be construed as a potential conflict of interest.

## Supplementary Material

Supplementary Material Data sheet provides individual information for SATv.

## Abbreviations

TPs: Tennis players
SATv: Subcutaneous adipose tissue volume
DXA: Dual-energy X-ray absorptiometry
MRI: Magnetic resonance imaging.

## References

[1] Ali O Cerjak D Kent JW Jr James R Blangero J Zhang Y. Obesity, central adiposity and cardiometabolic risk factors in children and adolescents: a family-based study. Pediatr Obes. (2014) 9:e58–62. 10.1111/j.2047-6310.2014.218.x 24677702 PMC4114214

[2] Malina RM Beunen GP Classens AL Lefevre J Vanden Eynde BV Renson R. Fatness and physical fitness of girls 7 to 17 years. Obes Res. (1995) 3:221–31. 10.1002/j.1550-8528.1995.tb00142.x 7627770

[3] Ara I Sanchez-Villegas A Vicente-Rodriguez G Moreno LA Leiva MT Martinez-Gonzalez MA. Physical fitness and obesity are associated in a dose-dependent manner in children. Ann Nutr Metab. (2010) 57:251–9. 10.1159/000322577 21150197

[4] Gonzalez-Alvarez C Ramos-Ibanez N Azprioz-Leehan J Ortiz-Hernandez L. Intra-abdominal and subcutaneous abdominal fat as predictors of cardiometabolic risk in a sample of Mexican children. Eur J Clin Nutr. (2017) 71:1068–73. 10.1038/ejcn.2017.28 28378850

[5] Boutcher SH. High-intensity intermittent exercise and fat loss. J Obes. (2011) 2011:868305. 10.1155/2011/868305 21113312 PMC2991639

[6] Baum T Cordes C Dieckmeyer M Ruschke S Franz D Hauner H. MR-based assessment of body fat distribution and characteristics. Eur J Radiol. (2016) 85:1512–8. 10.1016/j.ejrad.2016.02.013 26905521

[7] Ferrauti A Weber K Struder HK. Effects of tennis training on lipid metabolism and lipoproteins in recreational players. Br J Sports Med. (1997) 31:322–7. 10.1136/bjsm.31.4.322 9429011 PMC1332569

[8] Calbet JA Moysi JS Dorado C Rodriguez LP. Bone mineral content and density in professional tennis players. Calcif Tissue Int. (1998) 62:491–6. 10.1007/s002239900467 9576975

[9] Sanchis-Moysi J Dorado C Arteaga-Ortiz R Serrano-Sanchez AJ Calbet JA. Effects of training frequency on physical fitness in male prepubertal tennis players. J Sports Med Phys Fitness. (2011) 51:409–16. 10.1055/s-0030-1248331 21904279

[10] Sanchis-Moysi J Dorado C Vicente-Rodriguez G Milutinovic L Garces GL Calbet JA. Inter-arm asymmetry in bone mineral content and bone area in postmenopausal recreational tennis players. Maturitas. (2004) 48:289–98. 10.1016/j.maturitas.2004.03.008 15207895

[11] Gwinup G Chelvam R Steinberg T. Thickness of subcutaneous fat and activity of underlying muscles. Ann Intern Med. (1971) 74:408–11. 10.7326/0003-4819-74-3-408 5552114

[12] Krotkiewski M Aniansson A Grimby G Bjorntorp P Sjostrom L. The effect of unilateral isokinetic strength training on local adipose and muscle tissue morphology, thickness, and enzymes. Eur J Appl Physiol Occup Physiol. (1979) 42:271–81. 10.1007/BF00423297 161225

[13] Mohr DR. Changes in waistline and abdominal girth and subcutaneous fat following isometric exercises. Res Q. (1965) 36:168–73. 10.1080/10671188.1965.10614676 14329702

[14] Noland M Kearney JT. Anthropometric and densitometric responses of women to specific and general exercise. Res Q. (1978) 49:322–8. 10.1080/10671315.1978.10615541 725301

[15] Olson AL Edelstein E. Spot reduction of subcutaneous adipose tissue. Res Q. (1968) 39:647–52. 10.1080/10671188.1968.10616592 5246969

[16] Kostek MA Pescatello LS Seip RL Angelopoulos TJ Clarkson PM Gordon PM. Subcutaneous fat alterations resulting from an upper-body resistance training program. Med Sci Sports Exerc. (2007) 39:1177–85. 10.1249/mss.0b0138058a5cb 17596787

[17] Sanchis-Moysi J Idoate F Olmedillas H Guadalupe-Grau A Alayon S Carreras A. The upper extremity of the professional tennis player: muscle volumes, fiber-type distribution and muscle strength. Scand J Med Sci Sports. (2010) 20:524–34. 10.1111/j.1600-0838.2009.00969.x 19602193

[18] Ireland A Maden-Wilkinson T Ganse B Degens H Rittweger J. Effects of age and starting age upon side asymmetry in the arms of veteran tennis players: a cross-sectional study. Osteoporos Int. (2014) 25:1389–400. 10.1007/s00198-014-2617-5 24531424

[19] Olmedillas H Sanchis-Moysi J Fuentes T Guadalupe-Grau A Ponce-Gonzalez JG Morales-Alamo D. Muscle hypertrophy and increased expression of leptin receptors in the musculus triceps brachii of the dominant arm in professional tennis players. Eur J Appl Physiol. (2010) 108:749–58. 10.1007/s00421-009-1281-5 20187280

[20] Walsh S Haddad CJ Kostek MA Angelopoulos TJ Clarkson PM Gordon PM. Leptin and leptin receptor genetic variants associate with habitual physical activity and the arm body composition response to resistance training. Gene. (2012) 510:66–70. 10.1016/j.gene.2012.08.020 22975643 PMC3500611

[21] Hu HH Chen J Shen W. Segmentation and quantification of adipose tissue by magnetic resonance imaging. MAGMA. (2016) 29:259–76. 10.1007/s10334-015-0498-z 26336839 PMC5206913

[22] Maughan RJ Abel RW Watson JS Weir J. Forearm composition and muscle function in trained and untrained limbs. Clin Physiol. (1986) 6:389–96. 10.1111/j.1475-097X.1986.tb00244.x 3742958

[23] Goldberg EK Fung EB. Precision of the hologic DXA in the assessment of visceral adipose tissue. J Clin Densitom. (2019). 10.1016/j.jocd.2019.03.005 [Epub ahead of print]. 30992223 PMC6754313

[24] Shuster A Patlas M Pinthus JH Mourtzakis M. The clinical importance of visceral adiposity: a critical review of methods for visceral adipose tissue analysis. Br J Radiol. (2012) 85:1–10. 10.1259/bjr/38447238 21937614 PMC3473928

[25] Azzi AJ Lafreniere AS Gilardino M Hemmerling T. Ultrasonography technique in abdominal subcutaneous adipose tissue measurement: a systematic review. J Ultrasound Med. (2019) 38:877–88. 10.1002/jum.14789 30208232

[26] Davis JN Gyllenhammer LE Vanni AA Meija M Tung A Schroeder ET. Startup circuit training program reduces metabolic risk in Latino adolescents. Med Sci Sports Exerc. (2011) 43:2195–203. 10.1249/MSS.0b013e31821f5d4e 21502883 PMC3480316

[27] Owens S Gutin B Allison J Riggs S Ferguson M Litaker M. Effect of physical training on total and visceral fat in obese children. Med Sci Sports Exerc. (1999) 31:143–8. 10.1097/00005768-199901000-00022 9927022

[28] Staiano AE Katzmarzyk PT. Ethnic and sex differences in body fat and visceral and subcutaneous adiposity in children and adolescents. Int J Obes. (2012) 36:1261–9. 10.1038/ijo.2012.95 22710928 PMC4129655

[29] Van Belle G Statistical Rules of Thumb. New York, NY: John Wiley & Sons (2011). p. 33.

[30] Kuczmarski RJ Ogden CL Guo SS Grummer-Strawn LM Flegal KM Mei Z 2000 CDC Growth Charts for the United States: methods and development. Vital Health Stat. (2002) 11:1–190. 12043359

[31] Ara I Vicente-Rodriguez G Jimenez-Ramirez J Dorado C Serrano-Sanchez JA Calbet JA. Regular participation in sports is associated with enhanced physical fitness and lower fat mass in prepubertal boys. Int J Obes Relat Metab Disord. (2004) 28:1585–93. 10.1038/sj.ijo.0802754 15303104

[32] Duke PM Litt IF Gross RT. Adolescents' self-assessment of sexual maturation. Pediatrics. (1980) 66:918–20. 7454482

[33] Sanchis-Moysi J Idoate F Alamo-Arce D Calbet JA Dorado C. The core musculature in male prepubescent tennis players and untrained counterparts: a volumetric MRI study. J Sports Sci. (2017) 35:791–7. 10.1080/02640414.2016.1189589 27238230

[34] Sanchis-Moysi J Idoate F Serrano-Sanchez JA Dorado C Calbet JA. Muscle hypertrophy in prepubescent tennis players: a segmentation MRI study. PLoS ONE. (2012) 7:e33622. 10.1371/journal.pone.0033622 22428074 PMC3302769

[35] Lee RC Wang Z Heo M Ross R Janssen I Heymsfield SB. Total-body skeletal muscle mass: development and cross-validation of anthropometric prediction models. Am J Clin Nutr. (2000) 72:796–803. 10.1093/ajcn/72.3.796 10966902

[36] Idoate F Ibanez J Gorostiaga EM Garcia-Unciti M Martinez-Labari C Izquierdo M. Weight-loss diet alone or combined with resistance training induces different regional visceral fat changes in obese women. Int J Obes. (2011) 35:700–13. 10.1038/ijo.2010.190 20820174

[37] Bates D Mächler M Bolker B Walker S Fitting linear mixed-effects models using lme4. J Stat Softw. (2015) 67:1–48. 10.18637/jss.v067.i01

[38] R Core Team. R: A Language and Environment for Statistical Computing. Vienna: R Foundation for Statistical Computing (2017).

[39] Faul F Erdfelder E Lang A-G Buchner A. G^*^Power 3: a flexible statistical power analysis program for the social, behavioral, and biomedical sciences. Behav Res Methods. (2007) 39:175–91. 10.3758/BF03193146 17695343

[40] Hubers M Geisler C Plachta-Danielzik S Muller MJ. Association between individual fat depots and cardio-metabolic traits in normal- and overweight children, adolescents and adults. Nutr Diabetes. (2017) 7:e267. 10.1038/nutd.2017.20 28481336 PMC5518802

[41] Kim JA Park HS. Association of abdominal fat distribution and cardiometabolic risk factors among obese Korean adolescents. Diabetes Metab. (2008) 34:126–30. 10.1016/j.diabet.2007.10.012 18289908

[42] Gonzalez-Ruiz K Ramirez-Velez R Correa-Bautista JE Peterson MD Garcia-Hermoso A. The effects of exercise on abdominal fat and liver enzymes in pediatric obesity: a systematic review and meta-analysis. Child Obes. (2017) 13:272–82. 10.1089/chi.2017.0027 28322576

[43] Davis CL Pollock NK Waller JL Allison JD Dennis BA Bassali R. Exercise dose and diabetes risk in overweight and obese children: a randomized controlled trial. JAMA. (2012) 308:1103–12. 10.1001/2012.jama.10762 22990269 PMC3487697

[44] Saelens BE Seeley RJ van Schaick K Donnelly LF O'Brien KJ. Visceral abdominal fat is correlated with whole-body fat and physical activity among 8-y-old children at risk of obesity. Am J Clin Nutr. (2007) 85:46–53. 10.1093/ajcn/85.1.46 17209176 PMC1858646

[45] Alberga AS Prud'homme D Kenny GP Goldfield GS Hadjiyannakis S Gougeon R. Effects of aerobic and resistance training on abdominal fat, apolipoproteins and high-sensitivity C-reactive protein in adolescents with obesity: the HEARTY randomized clinical trial. Int J Obes. (2015) 39:1494–500. 10.1038/ijo.2015.133 26202452

[46] Lee S Bacha F Hannon T Kuk JL Boesch C Arslanian S. Effects of aerobic versus resistance exercise without caloric restriction on abdominal fat, intrahepatic lipid, and insulin sensitivity in obese adolescent boys: a randomized, controlled trial. Diabetes. (2012) 61:2787–95. 10.2337/db12-0214 22751691 PMC3478522

[47] Pino MF Parsons SA Smith SR Sparks LM. Active individuals have high mitochondrial content and oxidative markers in their abdominal subcutaneous adipose tissue. Obesity. (2016) 24:2467–70. 10.1002/oby.21669 27804230

[48] Huang TT Johnson MS Figueroa-Colon R Dwyer JH Goran MI. Growth of visceral fat, subcutaneous abdominal fat, and total body fat in children. Obes Res. (2001) 9:283–9. 10.1038/oby.2001.35 11346669

[49] Arner P Kriegholm E Engfeldt P Bolinder J. Adrenergic regulation of lipolysis *in situ* at rest and during exercise. J Clin Invest. (1990) 85:893–8. 10.1172/JCI114516 2312732 PMC296507

[50] Heinonen I Bucci M Kemppainen J Knuuti J Nuutila P Boushel R. Regulation of subcutaneous adipose tissue blood flow during exercise in humans. J Appl Physiol. (2012) 112:1059–63. 10.1152/japplphysiol.00732.2011 22223450

[51] Heinonen I Kemppainen J Kaskinoro K Knuuti J Boushel R Kalliokoski KK. Capacity and hypoxic response of subcutaneous adipose tissue blood flow in humans. Circ J. (2014) 78:1501–6. 10.1253/circj.CJ-13-1273 24759795

[52] Walker IV Smith CR Davies JH Inskip HM Baird J Methods for determining pubertal status in research studies: literature review and opinions of experts and adolescents. J Dev Orig Health Dis. (2019) 17:1–20. 10.1017/S2040174419000254 31204632

[53] Lundbom J Bierwagen A Bodis K Szendrodi J Kaprio J Rissanen A. Deep subcutaneous adipose tissue lipid unsaturation associates with intramyocellular lipid content. Metabolism. (2016) 65:1230–7. 10.1016/j.metabol.2016.05.010 27506730

